# Salmagundi

**Published:** 1886-03

**Authors:** 


### SALMAGUNDI.

EDITED BY E. G. BETTY, D.D.S.

Mr. Ground-hog failed to find his shadow – it was possibly lost in the mud.

PROFESSOR HUXLEY has accepted a pension of $1,500 per annum from the British Government.

---

CIGARETTES made of equal parts of tobacco and coca leaves are said to give great relief to the sufferer from asthma and hay fever.

---

The *London Lancet* says that, "If a man can keep his teeth until after middle age, he may generally count on keeping them to the end of his life."

---

JOHN B. GOUGH, the great temperance orator died February 18th in Frankford, Pa. His last words were "Young man, make your record clear."

---

MILLER has isolated from the secretions of the human mouth twenty-five different kinds of bacteria; of these, twelve are cocci and thirteen bacilli and bacteria.

---

THE editorial tilt between the *Independent Practitioner* and the *Items of Interest* on "a too liberal use of italics," with other things of note, is still on the tapis.

---

THE *Boston Post* says:—Music is the sound which one's children make as they romp through the house. Noise is the sound which other people's children make under the same circumstances.

---

ENGLISH doctors, accused by hysterical women of attempted assault, are now described by the London medical press as being "Bradleyed," that is, falsely charged, as Dr. Bradley is believed to have been.—*Clipping.*

---

THE *Canadian Pharmaceutical Journal* describes a novel form of stimulant, consisting of cocoa, tea, coffee, and cinchona, put up in plugs like tobacco. The unwise users of this compound are more considerate than tobacco chewers—they swallow the juice.—*Exchange.*

---

A WRITER in the *British Medical Journal* advises people to be careful not to slice up a pine-apple with the same knife they use in peeling it, as the rind contains an acrid organic substance which is likely to cause a swollen mouth and sore lips. In Cuba salt is used as an antidote for the poison of pine-apple peel.—*Clipping.*

---

LITTLE EDITH.—"Mr. Sapley, why does my sister Clara always pray when you come to see her?"

"Surely she doesn't. What do you mean?"

"Why, every time you come here and the servant comes up to the library to say you are in the parlor, Clara just shrugs her shoulders and says, 'O, Lord.'"

---

AKRON, O.—Roland C. Foust, the proprietor of a meat market, cut his hand badly ten days ago. His arm swelled, blood poisoning ensued, and this morning he died. He weighed 200 pounds, and was one of the most robust men in the city. The story current that in an altercation he struck his fist against his opponent's tooth, is denied by his family, but is generally credited.—*Daily Paper.*

---

OUR friend Dr. H. M. Reid, of Minneapolis, spent a week or two in Cincinnati last month, his estimable wife accompanying him on his visit. The Doctor has grown to immense and magnificent proportions, a fitting commentary on the quality and quantity of good "feed" in the great Northwest. Our only regret is that he could not remain long enough to attend the meeting of the M. V. D. A.

---

MR. WM. HERN, of London, Eng., recommends as a root-filling for dead teeth that have undergone conservative treatment wax and iodoform. The wax is to be melted on a heated slab and then about one half its bulk of iodoform incorporated with it. A wisp of cotton-wool is steeped in the warm mixture and carefully carried to its place with a suitable broach; about one half of the canal is to be filled with this, the balance with gutta-percha.

It appears to us that paraffine would be a better material than wax, as it possess many advantages over the latter.

---

THE *Independent Practitioner* for February in its "Current News and Opinion" quotes Prof. Virchow as saying: "No specialty can really flourish which cuts itself entirely off from the great body of the science," and then adds: "How is it when the science cuts itself off from the specialty?"

This is very amusing when we remember that Dr. Barrett proposed (and it was resolved) "That we as members of the dental profession deem it inexpedient to recommend the organization of a section of dental and oral surgery in the International Medical Congress of 1887, under the present circumstances." It also reminds us of a story told about Dr. Watt when called to task for something he had stated upon the floor of the American Dental Association "that it did not accord with the text-books." "So much the worse for the text-books," he replied.

---

THE announcement is made that Dr. Harlan, of Chicago, is a victim of insomnia and has sailed for Europe to get the benefit of sea breezes and a change of scene. The following clipping from a review of a work on "Brain Rest" is interesting: "A revised and enlarged edition has just been published of Dr. J. Leonard Corning's little book on "Brain Rest." The doctor gives much attention to that grave symptom, insomnia, and rules for removing it, thus preventing the train of ills leading to a break-down His remedy is not a trip to Europe "to be assailed by a countless array of petty extortions." A tranquil mind, an early hour for retiring, and a yielding to the first drowsy impulse in the evening are necessary conditions for the sufferer.

Dr. Corning has a plan for temporarily compressing the carotids in order to reduce the blood-flow to the brain, concerning which he makes a favorable report." The work is published by G. P. Putnam's Sons, New York.

